# Expanded tibiae enhance male mating success in the damselfly *Platycnemis phyllopoda*

**DOI:** 10.1016/j.isci.2026.116759

**Published:** 2026-07-10

**Authors:** Ilju Yang, Chang S. Han

**Affiliations:** 1 Department of Biology, Kyung Hee University, Seoul 02447, Korea; 2 Korea Institute of Ornithology, Kyung Hee University, Seoul 02447, Korea

**Keywords:** visual signal, leg, ornament, damselfly, tibia, *Platycnemis phyllopoda*

## Abstract

In insects, elaborate male secondary sexual traits have evolved through sexual selection driven by visual signals. In odonates, such displays typically feature wing pigmentation or flight behavior; however, investigations into non-wing morphological ornaments remain relatively rare. To address this gap, we investigated the damselfly *Platycnemis phyllopoda*, in which males possess conspicuously expanded, white mid and hind tibiae. Our findings revealed that males perform sequential courtship displays utilizing their UV-reflective tibiae. Field monitoring demonstrated that males with larger hind tibial areas achieved significantly higher mating success; however, they exhibited lower resighting frequencies and shorter observation spans, suggesting potential survival costs or increased dispersal. Combined with positive allometry, these results indicate that the expanded tibiae may function as sexually selected ornaments. Ultimately, opposing selection acting on these structures likely constrains their evolutionary exaggeration, highlighting the complex interplay of selections shaping tibial morphology in odonates.

## Highlights

• Male *Platycnemis phyllopoda* perform courtship through UV-reflective expanded tibiae
• Males with larger hind tibiae achieve higher pairing success
• Larger tibiae correlate with lower resighting and shorter observation spans
• Expanded tibiae show positive allometry

## Graphical abstract

**Figure:**
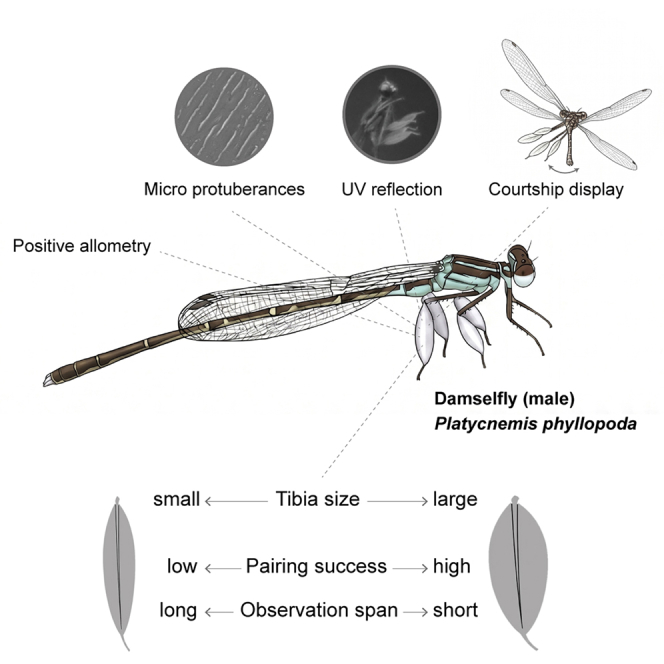

## Teaser

Zoology; Entomology; Evolutionary biology

## Introduction

In insects, numerous elaborate male traits have evolved through sexual selection,^1^^,^^2^^,^^3^^,^^4^^,^^5^^,^^6^^,^^7^^,^^8^^,^^9^ despite the costs associated with bearing them.^10^^,^^11^ These conspicuous secondary sexual traits may arise either via mate choice—conferring direct or indirect benefits to choosers^1^—or through advantages in intrasexual competition.^4^^,^^5^ Visual signals, in particular, are prominent drivers of mating success across various taxa. For example, male beetles with larger horns achieve greater success in both territorial contests and securing copulations.^4^^,^^12^ Conversely, some visual traits function exclusively in mate attraction rather than in agonistic interactions. For instance, the conspicuous wing coloration found in male butterflies serves primarily as a sexual signal rather than mediating intrasexual competition or species recognition.^13^^,^^14^^,^^15^^,^^16^

In the order Odonata, males often exhibit visually conspicuous secondary sexual traits,^17^^,^^18^ likely because odonates possess highly developed visual systems that are sensitive not only to the visible spectrum but also to ultraviolet (UV) wavelengths.^19^^,^^20^ Distinctive patterns or structures on the wings, abdomen, and legs presumably function as ornaments to attract mates, armaments to deter rivals, or both.^21^ To date, research has predominantly focused on wing traits and their effects on fitness. For example, male *Hetaerina americana* with larger red wing spots achieve higher mating success.^22^ Territorial *Pachydiplax longipennis* males with darker wings suffer fewer aggressive attacks and win more contests.^23^ Similarly, larger wing patches in *Libellula luctuosa* confer advantages in both territorial competition and mating.^24^ Furthermore, wing UV reflection in *Mnesarete pudica* appears to function as a visual signal.^25^ While *M. pudica* males generally elicit aggressive responses from rivals and sexual interest from females, UV-reduced males fail to evoke such reactions.^25^ However, conspicuous wing traits are subject to trade-offs, being constrained by factors such as thermoregulation^26^^,^^27^ and predation risk.^28^ Consequently, the elaborate wing ornamentation observed in those species likely reflects a balance where reproductive benefits outweigh survival costs.

**Figure 1:**
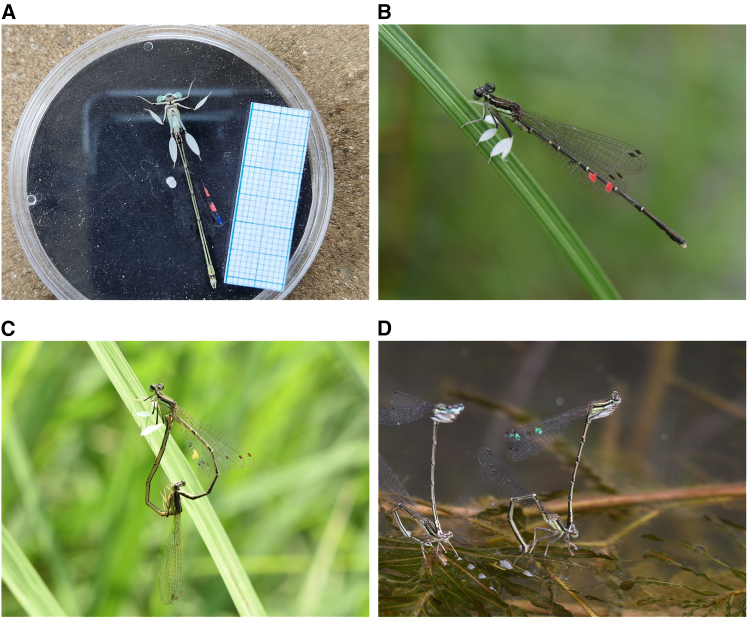
Male *Platycnemis phyllopoda* marked with colored dots (A) Representative photograph used for the measurement of abdomen length and hind tibial area. (B–D) Marked individuals observed during the post-release tracking period: (B) solitary male, (C) male *in copula*, and (D) male guarding a female during oviposition.

In contrast to wing traits, conspicuous leg structures in male odonates have received relatively little attention (Table 1), despite appearing to play similar roles in sexual selection.^47^^,^^48^ For example, exaggerated and brightly colored tibiae occur predominantly in the genus *Platycypha* (Chlorocyphidae) and the subfamily Platycnemidinae (Platycnemididae) of damselflies.^31^^,^^49^ The emergence of these tibiae ornaments across such phylogenetically distinct families suggests convergent evolution driven by strong, visually mediated sexual selection. This potentially represents a macroevolutionary shift away from the wing pigmentation commonly used by other odonate lineages. In male *Platycypha caligata*, the tibiae of all six legs are laterally expanded; the color of anterior surface is white, whereas the posterior surface is red.^48^ Experimental reversal of coloration between these surfaces was found to disrupt both territorial defense and mating success.^48^ In addition, absolute tibia length was positively associated with mating frequency and duration.^47^ Similar to *Platycypha caligata*, other species in the genus *Platycypha* possess laterally expanded tibiae with distinct anterior and posterior surfaces. In contrast, species in the subfamily Platycnemidinae exhibit anteroposteriorly expanded tibiae with distinct outer and inner surfaces, predominantly on the mid and hind legs (Figure 1B). The genus *Platycnemis* in the subfamily Platycnemidinae comprises 11 species,^50^ eight of which exhibit feather-like tibial expansions,^42^^,^^43^ with substantial interspecific variation in the extent of tibial expansion.^51^ Fossil evidence from the extinct *Yijenplatycnemis huangi* (c. 100 mya), a member of the family Platycnemididae, indicates that tibial expansion had already evolved in this lineage by the mid-Cretaceous.^37^ In a biogeographic context, *Platycnemis phasmovolans*, found in Southeast Asia near the fossil locality of *Y. huangi*, exhibits the most extensively expanded tibiae.^38^ Conversely, *Platycnemis* species in Europe and East Asia exhibit comparatively reduced tibial expansion. From a comparative phylogenetic perspective, this biogeographic pattern suggests that while the trait is ancient and evolutionarily conserved, the extreme exaggeration seen in tropical lineages may have undergone secondary reduction in temperate regions due to opposing selection or high fitness costs. Given that *Platycnemis* species retain remarkably enlarged tibiae—rare among other odonates—it is, therefore, essential to investigate the potential fitness benefits of this trait to fully understand its evolution.

**Table 1:** Tibial morphology and tibia-based courtship behaviors of species in the genera *Platycypha* (Chlorocyphidae) and *Platycnemis* (Platycnemididae), together with other species discussed in the text

Species	Hind tibial morphology	Hind tibial area (mm^2^)	Hindwing length (mm)	Tibial use in courtship	References
**Chlorocyphidae**					

*Platycypha auripes*	laterally expanded; white (anterior), yellow (posterior)	>2.7	–	?	Pinhey^29^; iNaturalist^30^
*Platycypha lacustris*	laterally expanded; white (anterior), red (posterior)	>2.7	–	?	Pinhey^29^; Dijkstra^31^
*Platycypha caligata*	laterally expanded; white (anterior), red (posterior)	2.7^b^	–	rapid vibration below the thorax	Robertson^32^; Tarboton^33^
*Platycypha angolensis*	laterally expanded; white (anterior), red (posterior)	∼2.7	–	?	Pinhey^29^; Kipping et al.^34^; iNaturalist^35^
*Platycypha fitzsimonsi*	laterally expanded; cream-colored (anterior), red (posterior)	1.6^b^	–	held together and swung from side to side	Pinhey^29^; Tarboton^33^; Robertson^36^
*Platycypha inyangae*	laterally expanded; white (anterior), red (posterior)	∼1.6	–	?	Pinhey^29^
*Platycypha pinheyi*	laterally expanded; white (anterior), red (posterior)	∼1.6	–	?	Pinhey^29^
*Platycypha amboniensis*	laterally expanded; orange	<1.6	–	?	Pinhey^29^
*Platycypha picta*	unexpanded; yellow	–	–	?	Pinhey^29^; Dijkstra^31^
*Platycypha rufitibia*	unexpanded; white (anterior), red (posterior)	–	–	?	Pinhey^29^; Dijkstra^31^
*Platycypha bamptoni*	unexpanded; white (anterior), black (posterior)	–	–	?	Kipping et al.^34^
*Chlorocypha consueta*	unexpanded; white (anterior), black (posterior)	–	–	held together beneath the thorax without visible movement	Robertson^36^
*Rhinocypha unimaculata*	unexpanded; white (anterior), black (posterior)	–	–	held together beneath the thorax without visible movement	Robertson^36^

**Platycnemididae**					

a*Yijenplatycnemis huangi*	posteriorly expanded; hyaline with two brown bands	13.8^b^	14.1	?	Zheng et al.^37^
*Platycnemis phasmovolans*	anteroposteriorly expanded	8.4^b^	19.0	?	Hamalainen^38^
*Platycnemis foliacea*	anteroposteriorly expanded	<8.4, >3.9	18.5	?	Hamalainen^38^; Hämäläinen^39^
*Platycnemis phyllopoda*	anteroposteriorly expanded	3.9	19.0	a sequential display involving tibial spreading, forward extension, and subsequent lateral movement	**This study**
*Platycnemis sasakii*	anteroposteriorly expanded	∼3.9	22.0	?	Hamalainen^38^
*Platycnemis latipes*	anteroposteriorly expanded	<3.9	20.0	not used in courtship	Yu and Cordero-Rivera^40^; Smallshire and Swash^41^
*Platycnemis pennipes*	anteroposteriorly expanded; black stripe (outer)	<3.9	21.0	?	Smallshire and Swash^41^; Kalkman^42^
*Platycnemis dealbata*	anteroposteriorly expanded	<3.9	–	?	Kalkman^42^
*Platycnemis acutipennis*	anteroposteriorly expanded; incomplete black stripe (outer)	<3.9	18.5	?	Smallshire and Swash^41^
*Platycnemis echigoana*	unexpanded; black (outer)	–	–	?	Asahina^43^; iNaturalist^44^
*Platycnemis kervillei*	unexpanded; blue pruinosity	–	–	?	Kiany et al.^45^
*Platycnemis subdilatata*	unexpanded; black stripe (outer)	–	–	?	iNaturalist^46^

In *Platycypha*, tibial expansion occurs in all six legs, whereas in *Platycnemis*, it is restricted to the mid and hind legs. Assessment of tibial morphology and area was based on the hind tibiae of mature males. Tibial colors are reported separately where the two surfaces differ, and as a single color when they are identical. Unless otherwise specified under tibial morphology, the tibia is white. Where precise area measurements were lacking, relative within-genus comparisons are provided. For species in the family Platycnemididae, mean hindwing length was additionally included from the literature as an approximate proxy for body size.

a An extinct species known only from the fossil record.

b Tibial areas were approximately estimated from published reference photographs using ImageJ software.

To determine whether tibial expansion in damselflies has evolved via sexual selection, it is highly informative to examine its “static allometry” (hereinafter referred to as allometry)—the proportional scaling of the trait relative to overall body size among conspecifics at the same developmental stage. While some structures scale directly with body size (isometry), sexually selected traits often scale disproportionately, exhibiting either positive or negative allometry.^52^^,^^53^^,^^54^^,^^55^^,^^56^ By quantifying these allometric slopes, evolutionary biologists can infer how individuals of varying sizes differently invest in specific body parts.^54^^,^^57^^,^^58^ This, in turn, provides critical insights into selection—particularly sexual selection—acting upon these ornamental traits,^53^^,^^54^^,^^59^^,^^60^^,^^61^ although recent studies caution against relying solely on allometric steepness to infer the strength of directional selection (e.g., see works by Bertin and Fairbairn^62^ and Shinohara et al.^63^). Moreover, depending on their specific function, the allometric slopes of sexually selected traits can vary significantly, ranging from strongly positive (e.g., weapon) to negative (e.g., genitalia or contact courtship traits).^52^^,^^53^^,^^56^^,^^64^^,^^65^ For example, in the case of visual ornaments that are displayed to females from a distance and serve as indicators of male quality (i.e., non-contact courtship trait), psychophysical principles (e.g., Weber’s law^66^^,^^67^) suggest that larger males must produce disproportionately larger ornaments to enable females to perceive the size difference and elicit a response. Consequently, positive allometry is generally predicted for the visually conspicuous expanded tibiae in damselflies. Nevertheless, this prediction is subject to certain functional and ecological caveats. If the ornament does not reflect underlying male quality but merely functions to stimulate females, it is expected to exhibit a less pronounced positive allometry.^53^ In addition, because damselfly legs are also essential for capturing and retaining prey,^68^ opposing natural selection associated with these foraging functions may constrain the evolution of positive allometry in this trait.

In this study, we provide the first detailed description of the courtship behavior and expanded tibial morphology of male *Platycnemis phyllopoda*, a damselfly species indigenous to East Asia. We further investigate, through field studies, whether these tibial traits are associated with male mating success. Males of *P. phyllopoda* possess white, feather-like tibial expansions on the mid and hind legs (Figure 1B). The expanded tibiae of *P. phyllopoda* are smaller than those of *P. phasmovolans* but broader than those of the European species *P. latipes* and *P. pennipes*, even though the overall body size of these species do not differ significantly (Table 1). Recent research has demonstrated that the expanded tibiae of male *P. phyllopoda* function as visual signals that facilitate sex recognition and reduce unnecessary male-male interactions during patrol flights.^69^ Although the use of these structures has also been observed in interactions with females,^69^ their potential role in sexual signaling and its effect on mating success remain unclear. We hypothesized that these expanded tibiae function as sexually selected ornaments enhancing mating success, given that males actively display these conspicuous structures during courtship flights (see results).

To test this, we first systematically described the male courtship sequence. We then characterized the fine-scale microstructures of the tibiae using scanning electron microscopy (SEM) and evaluated their potential UV reflectance. In particular, a previous study demonstrated that the expanded tibiae of the related species *P. sasakii* exhibit highly conspicuous UV reflectance compared with other body regions.^70^ Given that UV-reflective ornamental traits (e.g., UV-reflective wings) are important in eliciting female sexual responses in Odonata,^25^ we predicted that the expanded tibiae of male *P. phyllopoda* play a similar role in enhancing male mating success. To empirically test this, we individually marked males in the field to assess the association between hind tibial area and mating success, while simultaneously analyzing the relationship between hind tibial area and both resighting frequency and observation span to assess potential costs. While elaborate secondary sexual traits offer clear reproductive advantages, they may also impose significant costs, such as increased predation risk or reduced survival. Understanding these potential trade-offs is essential for elucidating the selection that shapes ornaments. Finally, to explore the potential selection acting on tibial expansion, we calculated the allometric slopes of tibial area. Because non-contact visual sexual signals, such as wing pigmentation, in damselflies often exhibits positive allometry,^71^ we predicted that tibial area, functioning as a non-contact visual sexual signal, would similarly be under directional sexual selection and exhibit positive allometry.

## Results

### Courtship behavior

**Figure 2:**
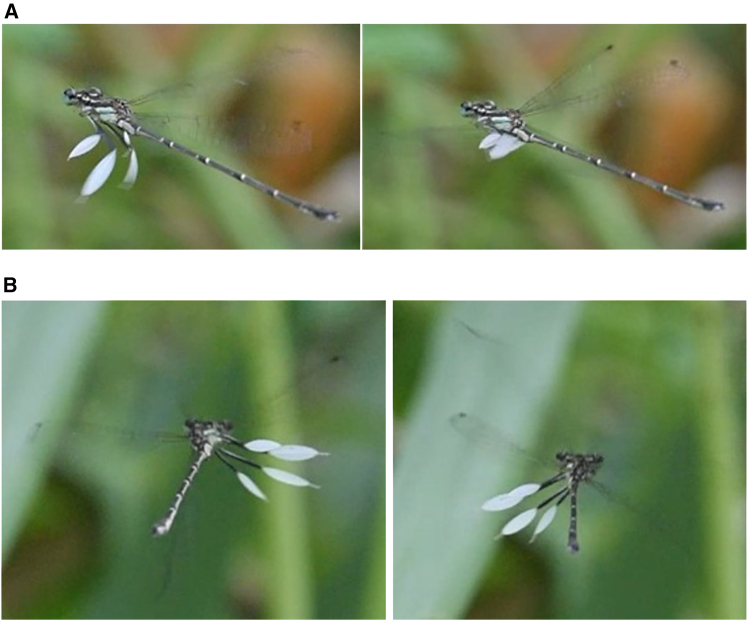
Courtship behavior of male *Platycnemis phyllopoda* (A) Courtship commences with a hovering display characterized by repeated spreading and folding of the mid and hind legs. As the male flies toward the female, the folding phase progressively diminishes, transitioning into a display where the legs are spread and extended forward. (B) In the final stage, upon approaching the female, the legs are maintained in a fully extended position beneath the thorax and swung laterally during the attempt at tandem formation.

We categorized the courtship sequence of male *P. phyllopoda* into four distinct stages (Videos S1 and S2), delineated on the basis of specific, stereotyped behavioral transitions exhibited by the males: (1) Upon detecting a female, the male initiated hovering flight and oriented his body toward her. (2) The male then commenced tibia-based courtship displays while hovering. During this stage, the mid and hind legs, bearing the expanded tibiae, were repeatedly spread and then fully folded beneath the thorax (Figure 2A). (3) As the male flew toward the female, these repeated movements persisted; however, the folding phase became progressively reduced. The display eventually transitioned into a pattern in which the legs were repeatedly spread, drawn together, and subsequently extended forward. (4) Upon closely approaching the female, the male maintained the legs in a fully extended position beneath the thorax and swung them laterally while attempting tandem formation (Figure 2B).

Throughout the entire courtship sequence, the forelegs were not involved in any of the displays. All five males that were recorded and analyzed exhibited this identical behavioral sequence during courtship. Although high-speed video recordings suitable for detailed slow-motion analysis were limited to five individuals due to the logistical challenges of filming in the field, this sequential display appears representative of the study population based on broader field observations (I. Yang, personal observation). During courtship, females occasionally avoided the male by arching the abdomen upwards at the onset of stage 3 (Video S1); in other instances, they remained stationary and accepted the tandem formation attempt (Video S2). Furthermore, when males persisted in courtship despite female escape attempts, females occasionally displayed aggressive behaviors.

Video S1. Courtship behavior of *Platycnemis phyllopoda*; in the final stage, the female rejects the male by arching her abdomen upwards

Video S2. Courtship behavior of *Platycnemis phyllopoda*; the female remains stationary and accepts the attempt at tandem formation

### Scanning electron microscopy of expanded tibiae

**Figure 3:**
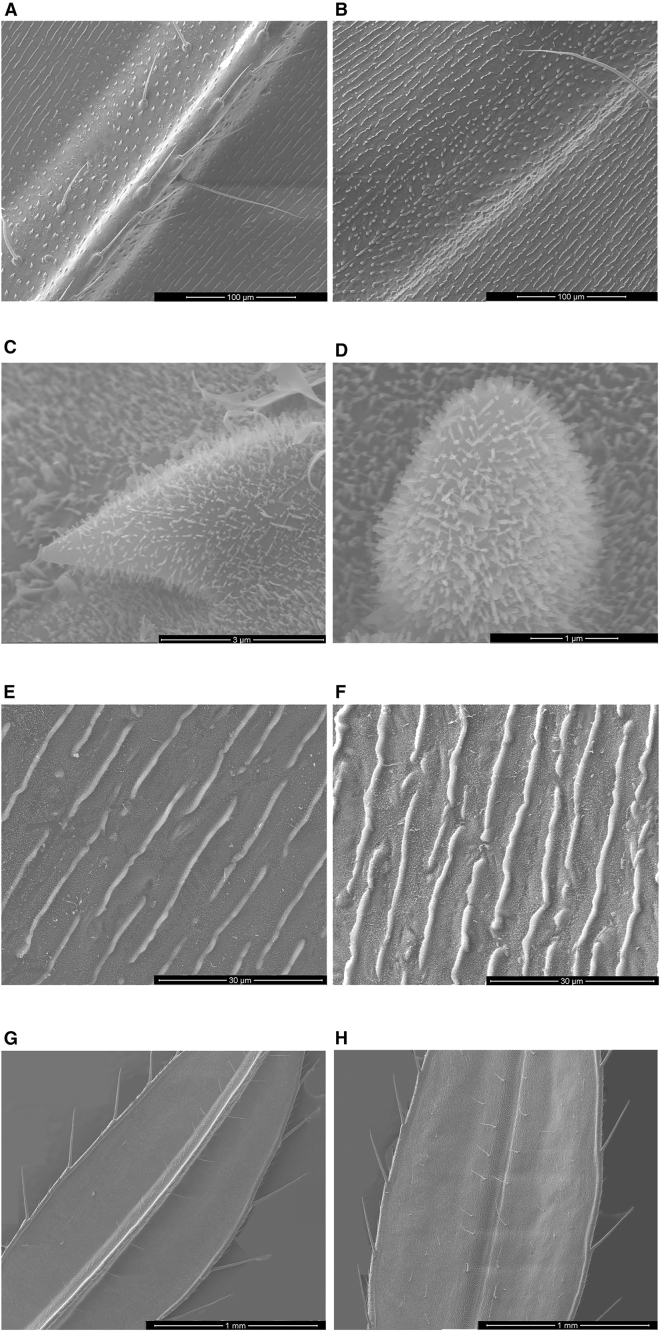
Field emission scanning electron microscopy images of the expanded tibia of male *Platycnemis phyllopoda* (A and B) Central regions of the outer and inner surfaces, respectively (×1,000). (C) Triangular protuberances in the central region of the outer surface (×50,000). (D) Circular protuberances in the central region of the inner surface (×80,000). (E and F) Expanded portions of the outer and inner surfaces, respectively (×5,000). (G and H) Overall morphology of the outer and inner tibial surfaces, respectively (×150). Scale bars are indicated in each part of the image.

The expanded tibiae of male *P. phyllopoda* were examined using field emission scanning electron microscope (FE-SEM). The tibia is laterally expanded along the median ridge and bears numerous minute protuberances (Figures 3A–3H). While the distribution patterns of micro-protuberances were similar between the mid and hind tibiae, distinct differences were observed between the outer and inner surfaces of the tibiae (Figures 3A–3F). In the central region, the outer surface exhibited scattered, isolated triangular protuberances with setae along the median ridge (Figures 3A and 3C). In contrast, the inner surface featured scattered, isolated circular protuberances and lacked setae along the median ridge (Figures 3B and 3D). In the expanded region, both surfaces possessed longitudinally arranged vertical protuberances and sparse setae, the latter being more abundant on the inner surface (Figures 3E–3H). The vertical protuberances were more numerous and densely spaced on the inner surface compared with the outer surface (Figures 3E and 3F).

**Figure 4:**
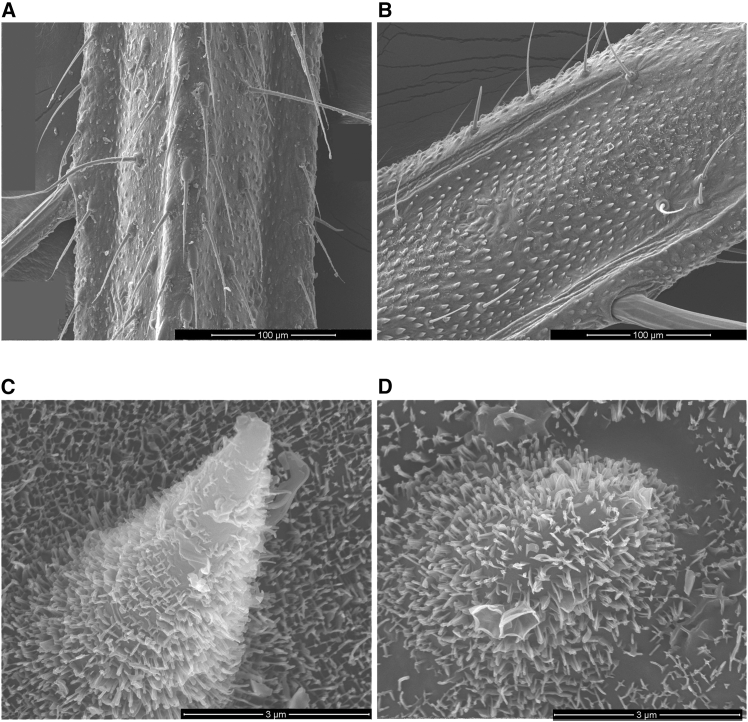
Field emission scanning electron microscopy images of the fore tibia of male *Platycnemis phyllopoda* (A and B) Proximal regions of outer and inner surfaces, respectively (×1,000). (C) Triangular protuberances in the central region of the outer and inner surfaces (×50,000). (D) Circular protuberances in the marginal region of the inner surface (×50,000). Scale bars are indicated in each part of the image.

Unlike the expanded regions of the mid and hind tibiae, the fore tibia lacked prominent, longitudinally arranged vertical protuberances. Instead, triangular and circular protuberances—akin to those in the central zones of the expanded tibiae—were predominantly restricted to the proximal portion (Figure 4). Specifically, the inner surface of this proximal area featured triangular protuberances in the central area and circular ones in the marginal zone (Figures 4B–4D). While vertical protuberances occurred between these structures, they were markedly diminished in size compared with those observed in the expanded regions of the mid and hind tibiae (Figures 3A, 3B, and 4B).

### Ultraviolet reflectance of expanded tibiae

**Figure 5:**
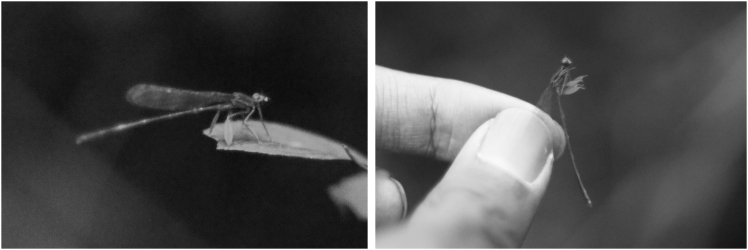
Ultraviolet reflectance of expanded tibiae in male *Platycnemis phyllopoda* UV-reflective areas appear white, whereas non-reflective areas appear black.

The expanded tibiae of male *P. phyllopoda* reflected UV light, whereas the remaining body regions including the central lines of the expanded tibia showed negligible UV reflectance (Figure 5). Furthermore, UV reflectance from the expanded tibiae was angle-dependent, appearing strongest when the tibiae were oriented parallel to the observer’s line of sight.

### Field experiment: Effects of tibia size on male mating success, resighting frequency, and observation span

**Figure 6:**
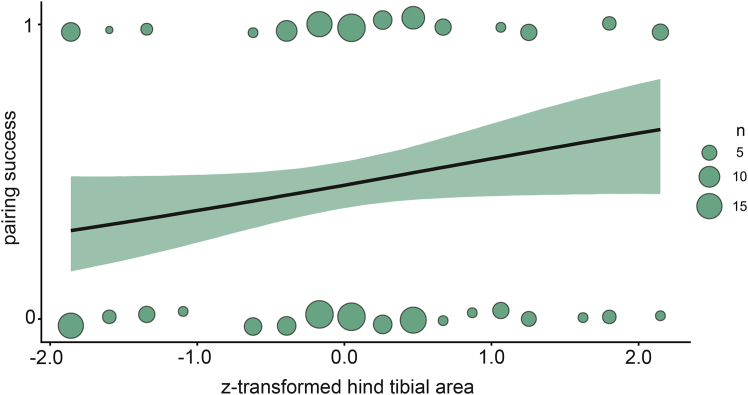
Relationship between hind tibial area and pairing success in male *Platycnemis phyllopoda* Pairing success was scored as a binary variable (1 = success, 0 = failure). The solid line represents the predicted probability from the model described in Table 2, with the surrounding shaded region indicating the 95% confidence interval. Individual data points show observed outcomes plotted against z-transformed hind tibial area; the size of each point scales with the number of observations (frequency).

Males with larger tibiae achieved significantly higher mating success (Table 2; Figure 6). Specifically, a one standard deviation increase in hind tibial area was associated with an approximate 81.9% increase in the probability of mating. This effect was independent of abdomen length, and neither weather conditions nor tibial damage significantly influenced mating success (Table 2). Furthermore, no significant interaction between hind tibial area and weather conditions was detected (Table 2). Although the observed mating frequency within the population tended to decline over the course of the observation period, this trend was not statistically significant (Table 2).

**Table 2:** Results of the generalized linear mixed-effects model examining the effects of weather conditions, hind tibial area, observation day, abdomen length, and tibia loss on mating success in male *Platycnemis phyllopoda*

	β (95% CI)	Odds ratio (95% CI)
Intercept	0.63 (−1.71, 2.98)	1.80 (0.17, 19.40)
Weather principal component	0.22 (−0.04, 0.48)	1.24 (0.94, 1.63)
Hind tibial area^a^	0.36 (0.04, 0.70)	1.47 (1.03, 2.10)
Weather PC ∗ hind tibial area	0.06 (−0.15, 0.27)	1.06 (0.85, 1.32)
Observation day^b^	−0.32 (−0.67, 0.02)	0.74 (0.52, 1.05)
Abdomen length^a^	−0.13 (−0.44, 0.18)	0.86 (0.61, 1.21)
Lost tibia^c^	−0.83 (−3.19, 1.53)	0.45 (0.04, 4.96)

Parameter estimates (β), odds ratios, and corresponding 95% confidence intervals (CIs) are shown.

a log transformed and z-standardized.

b z-standardized.

c coded as 0 = intact, 1 = lost.

**Figure 7:**
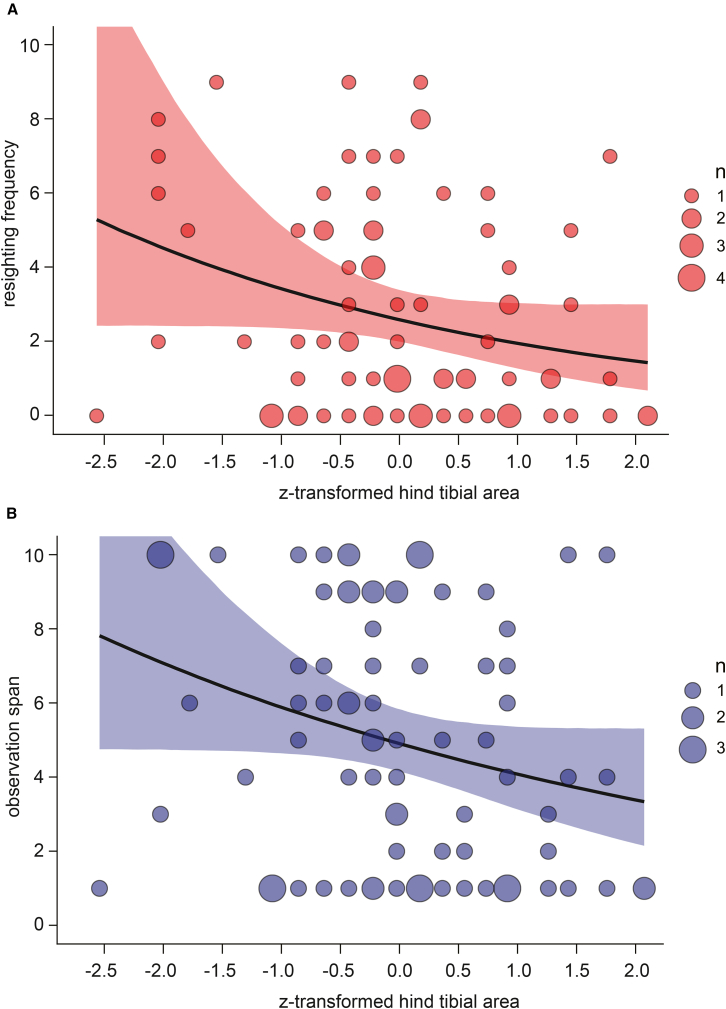
Hind tibial area, resighting frequency, and observation span Relationships between hind tibial area and (A) post-marking resighting frequency and (B) observation span in male *Platycnemis phyllopoda*. Predicted relationships (solid lines) were derived from generalized linear models adjusted for body length, with shaded areas indicating 95% confidence intervals. Individual data points (*n* = 83) are shown against z-transformed hind tibia area; point diameter reflects the frequency of overlapping observations.

Larger hind tibial area was also associated with a significantly lower frequency of post-marking observations (β [95% CI] = −0.23 [−0.46, −0.01], Figure 7A) and a shorter observation span (β [95% CI] = −0.24 [−0.47, −0.01], Figure 7B). In contrast, abdomen length was unrelated to the frequency of observations following marking (β [95% CI] = −0.01 [−0.23, 0.21]) or the observation span (β [95% CI] = −0.02 [−0.25, 0.22]).

### Allometric slope

The non-sexual traits—lengths of the fore tibia, mid tibia, hind tibia, mid femur, and hind femur—showed isometry (Table 3). Among the sexual traits, the mid tibial area showed strongly positive allometry and the hind tibial area tended to exhibit positive allometry (Table 3).

**Table 3:** Allometric slopes for non-sexual traits (lengths of the fore tibia, mid tibia, hind tibia, mid femur, and hind femur) and sexual traits (mid and hind tibial areas) in male *Platycnemis phyllopoda*

Body size proxy: abdomen	SMA slope (95% CI)	Body size proxy: hindwing	SMA slope (95% CI)	*n*
Hindwing	1.03 (0.84, 1.26)	abdomen	0.97 (0.79, 1.19)	47
Fore tibia	1.02 (0.80, 1.32)	fore tibia	1.00 (0.79, 1.27)	46
Mid tibia	0.99 (0.77, 1.28)	mid tibia	0.97 (0.76, 1.23)	47
Hind tibia	1.05 (0.80, 1.37)	hind tibia	1.02 (0.79, 1.32)	47
Mid femur	1.09 (0.85, 1.39)	mid femur	1.06 (0.84, 1.34)	47
Hind femur	0.79 (0.59, 1.05)	hind femur	0.77 (0.58, 1.03)	37
Mid tibial area	1.67 (1.36, 2.06)	mid tibial area	1.63 (1.31, 2.03)	47
Hind tibial area	1.27 (0.99, 1.62)	hind tibial area	1.23 (0.97, 1.57)	47

Slopes were estimated using standardized major axis (SMA) regressions, with abdomen and hindwing length serving as body size proxies. Area values were square-root transformed prior to analysis to ensure dimensional consistency. Values represent slope estimates with 95% confidence intervals (CIs), and sample sizes (*n*) are provided for each trait.

## Discussion

Our results suggest that the expanded tibiae of male *P. phyllopoda* appear to function in visually mediated courtship directed toward females and could potentially confer significant reproductive benefits. Observations of courtship behavior revealed that males perform a specific sequence of displays involving the mid and hindlegs, both of which bear the expanded tibiae. Furthermore, these structures exhibited pronounced UV reflectance compared with other body regions (Figure 5), a pattern consistent with the expanded tibiae of *Platycnemis foliacea* (currently *P. sasakii*)^70^ and other UV-reflective ornaments described in damselflies.^25^ While the physical mechanism underlying this reflectance remains unclear, SEM observations suggest that it may arise from the unique microstructures on the expanded tibiae, such as longitudinally arranged vertical protuberances (Figures 3 and 4). These reflective properties are hypothesized to facilitate visual signaling during male-female interactions, although further empirical investigation, especially concerning female perception, is required to establish their functional efficacy. In line with their potential role in sexual selection, our field experiments involving individual marking and monitoring over a 10-day period demonstrated that male mating success increased with hind tibial area. Because *P. phyllopoda* exhibits negligible territorial behavior,^69^ these structures are unlikely to function in male-male competition. These findings suggest that the expanded tibiae appear to function primarily as sexually selected ornaments.

However, the ultimate fitness consequence of bearing larger tibiae remain ambiguous. We found a negative relationship between tibial area and both local resighting frequency and observation span. While this could tentatively be interpreted as a potential survival cost associated with larger ornaments, such a conclusion must be approached with caution. It is equally plausible that this lower resighting probability is driven by alternative factors, such as size-biased dispersal—whereby males with larger tibiae exhibit greater mobility—or reduced detectability within the habitat. Consequently, although our findings indicate that the expanded tibiae of *P. phyllopoda* likely provide a mating advantage, determining whether the reduced local residency of males with larger tibiae reflects a true viability cost or merely a shift in spatial behavior will require further investigation.

The courtship behavior of male *P. phyllopoda* shares characteristics with displays observed in the family Chlorocyphidae, yet appears to be distinguished by its greater complexity involving multiple distinct movements (Table 1). Tibia-based courtship displays are well-documented in Chlorocyphidae, although specific patterns vary among species.^36^ For instance, males of *Platycypha caligata* court by independently vibrating their expanded tibiae, whereas males of *Platycypha fitzsimonsi*, which possess smaller tibiae, hold their legs together and swing them laterally.^36^ In contrast*, Chlorocypha consueta* and *Rhinocypha unimaculata*, which feature white but non-expanded tibiae, display by holding the legs static beneath the thorax.^36^ In *P. phyllopoda*, which possesses broader tibiae than *Platycypha caligata* and *Platycypha fitzsimonsi* (Table 1), tibia-based courtship comprises two clearly distinguishable components, as described by Xin Yu et al.^69^ in interactions with females: the repeated spreading and folding of the markedly expanded tibiae, followed by the lateral swinging of the legs held together (Figure 2; Videos S1 and S2). These movements are organized into discrete stages, forming a sequential courtship display. In Chlorocyphidae, males typically defend fixed territories and court females within those bounds as they inspect oviposition sites.^32^ By contrast, *P. phyllopoda* is non-territorial; males actively search for females and initiate courtship immediately upon detection. This difference in ecological and behavioral context may underlie the evolution of the staged, sequential courtship display seen in *P. phyllopoda*. Comparative evidence from Chlorocyphidae suggests that species with more extensively expanded tibiae tend to exhibit more complex courtship behaviors (Table 1), implying that a larger tibial area facilitates more active or elaborate use of the legs. Consistent with this pattern, tibiae are used actively during courtship in *P. phyllopoda*, whereas no tibia-based courtship behavior has been reported in the congeneric *P. latipes*,^40^ a species with significantly less expanded tibiae. Taken together, even within the genus *Platycnemis*, interspecific variation in the extent of tibial expansion may be associated with the diversification of courtship behavior. Although our observations of courtship behavior are exploratory, we suggest that the tibial morphology and associated courtship behaviors in Chlorocyphidae and *Platycnemis* (Platycnemididae) serve similar functional roles in courtship, even if they have evolved independently.

Beyond courtship, the expanded tibiae in *P. phyllopoda* are also used during patrol flights, although this may not represent the primary selective driver of tibial expansion. In the congeneric species *P. latipes*, the tibiae are less expanded than in *P. phyllopoda*, yet more than half of the abdomen is white. Rather than displaying their tibiae during patrol flights, *P. latipes* males exhibit a characteristic zigzag flight pattern that enhances the visibility of the white abdomen, a mechanism suggested to reduce male-male interference by facilitating sex recognition.^40^ In contrast, *P. phyllopoda* possesses a predominantly black abdomen but features extensively expanded white tibiae. Although a pronounced zigzag flight is absent, males of *P. phyllopoda* frequently articulate their legs when searching for females or encountering other males, thereby flashing the expanded tibiae.^69^ Such displays similarly function to signal male identity and potentially reduce misdirected interactions. Taken together with previous findings, our results suggest that the expanded tibiae of *P. phyllopoda* serve dual functions. Although it remains unclear which function evolved first or is more important, tibiae are not used in direct competition, as males typically ignore other males displaying them.^69^ In contrast, our results demonstrate that a larger hind tibial area confers a clear mating advantage. These patterns suggest that the role of tibiae in male-male recognition may be secondary, with this function instead being performed by the conspicuous white abdomen in the congeneric species *P. latipes*.

In conjunction with previously described ornaments in odonates,^22^^,^^23^^,^^24^^,^^25^^,^^48^ our findings indicate that expanded tibiae in *P. phyllopoda* males are associated with mating success and likely play a role during courtship interactions. The allometric slopes of mid and hind tibial area, particularly those of the mid tibia, indicate positive allometry, whereas non-sexual traits show isometry. These results suggest that the expanded tibiae in this species probably evolved via female mate choice, although the precise evolutionary mechanisms driving their expansion require further clarification. For example, conspicuous tibiae and their associated displays might attract female attention by exploiting pre-existing visual sensitivities in females.^19^^,^^72^ Alternatively, tibial area may function as an honest indicator of male condition (i.e., the “good genes” hypothesis^73^^,^^74^). Examining how the tibial area develops under varying rearing conditions would enable us to robustly assess the condition dependence of this ornament. Furthermore, this extensive expansion might be driven by a genetic correlation between the male trait and the female preference for it (i.e., runaway selection^75^^,^^76^^,^^77^). Beyond testing these hypotheses, investigating the potential selective constraints acting against extreme tibial exaggeration will be crucial for a comprehensive understanding of this trait’s evolution. Moreover, the association between extensive tibial expansion and complex, sequential courtship behavior in the non-territorial *P. phyllopoda* might offer valuable insights into the coevolution of morphological and behavioral sexual traits. Ultimately, future phylogenetic studies examining both tibial morphology and its functional use across courtship and other contexts will be key to unraveling how the dynamic interplay between sexual selection favoring ornamentation and opposing selection constraining its expression shapes the evolution of complex reproductive strategies in damselflies.

### Limitations of the study

While our field experiment captured the peak reproductive season, the study was limited by a relatively short observation period (10 days) and a lack of data on male age and social history. These unmeasured variables prevent us from unequivocally confirming that expanded tibiae evolved solely via sexual selection. Therefore, longitudinal studies tracking individuals from emergence are necessary to precisely assess lifetime reproductive benefits. In addition, as our study was restricted to pairing success, future work should extend to measuring realized reproductive success beyond the pre-mating stage.

Moreover, the negative relationship between tibia size and resighting frequency might be driven by size-biased dispersal or different microhabitat use rather than solely by survival costs incurred by larger tibiae. While our study site was selected for its isolation to minimize emigration and maximize resighting probability, and *P. phyllopoda* is known to exhibit high site fidelity,^69^ the possibility of dispersal cannot be entirely excluded. Therefore, it is plausible that some individuals absent from subsequent monitoring may have emigrated rather than died. Future studies employing more sophisticated demographic modeling, such as the Cormack-Jolly-Seber framework, are required to disentangle these specific parameters.

In addition, rigorous testing is needed to evaluate the functional efficacy of UV reflectance from the expanded tibiae and its effect on female behavior. Although simple UV photography demonstrates the presence of UV reflectance, obtaining precise spectrophotometric reflectance curves is essential to mechanistically understand how these signals operate under sexual selection. By quantifying reflectance across specific wavelengths, receiver-based models unique to female damselfly visual sensitivities can simulate exactly how these UV signals are perceived. Additionally, it is critical to assess this signal against the visual noise of the natural habitat. Generating reflectance curves for both the expanded tibiae and diverse natural backgrounds (such as green leaves, brown mud, and water surfaces) enables the precise calculation of visual contrast.

Finally, we acknowledge that our current findings are limited to correlational patterns. To address this limitation, future experimental studies involving the artificial manipulation of tibial area or UV reflectance and estimating female responses are necessary to assess the direct effect of tibiae on male mating success. By comparing pairing success across these manipulated treatments, researchers could definitively confirm whether the expanded tibiae themselves serve as the primary target of mate choice, independent of other correlated male traits. In addition, if we can quantify female responses to males varying in tibial area (e.g., latency to copulation and rejection frequency prior to acceptance) from the field or lab-controlled environment, it would be ideal to test mechanisms of female mate choice related to male tibiae.

## Resource availability

### Lead contact

Requests for further information and resources should be directed to and will be fulfilled by the lead contact, Chang S. Han (hcspol@gmail.com).

### Materials availability

This study did not generate new unique reagents.

### Data and code availability

• All data have been deposited at Figshare and are publicly available as of the date of publication at https://doi.org/10.6084/m9.figshare.31230673.
• All original code has been deposited at Figshare and is publicly available as of the date of publication at https://doi.org/10.6084/m9.figshare.31230673.
• Any additional information required to reanalyze the data reported in this paper is available from the lead contact upon request.

## Acknowledgments

We thank the members of the Evolutionary Ecology Laboratory for their help with collecting adult damselflies. This work was supported by the National Research Foundation of Korea (NRF) grants funded by the Korea government (NRF-2022R1C1C1004303, RS-2024-00405751, and RS-2025-16067311 to C.S.H.) and the Global-Learning & Academic Research Institution for Master’s/PhD students and Postdocs (G-LMAP) Program of the NRF grant funded by the Ministry of Education (RS-2025-25442355 to C.S.H.).

## Author contributions

Conceptualization, I.Y. and C.S.H.; data curation, I.Y.; formal analysis, I.Y. and C.S.H.; funding acquisition, C.S.H.; investigation, I.Y.; methodology, I.Y. and C.S.H.; project administration, C.S.H.; visualization, I.Y. and C.S.H.; writing – original draft, I.Y. and C.S.H.; writing – review and editing, I.Y. and C.S.H.

## Declaration of interests

The authors declare no competing interests.

## Declaration of generative AI and AI-assisted technologies in the writing process

During the preparation of this work, the authors used Gemini and ChatGPT to assist with English grammatical corrections. Additionally, AI tools were employed to design and render the graphical abstract illustration. After using these tools, the authors reviewed and edited the content as needed and take full responsibility for the content of the publication.

## STAR★Methods

### Key resources table

**Table:** 

REAGENT or RESOURCE	SOURCE	IDENTIFIER
**Experimental models: Organisms/strains**		

*Platycnemis phyllopoda*	Details in method details section below	–

**Software and algorithms**		

ImageJ (version 1.54g)	Schindelin et al.^78^	https://imagej.net/ij/download.html
R programming language	R Core Team, 2025	https://www.r-project.org/
R-Studio (v. 4.5.0)	Posit PBC	https://posit.co/download/rstudio-desktop

**Deposited data**		

Data and original code	This paper	https://doi.org/10.6084/m9.figshare.31230673

**Other**		

Nikon D810	Nikon	https://www.nikon.com/
60 mm Lens	Nikon	https://www.nikon.com/
150 mm Lens	Irix	https://irixlens.com/
FE-SEM; Nova Nano SEM 200	FEI	https://www.thermofisher.com/kr/en/home.html
Canon EOS 5D Mark III	Canon	https://global.canon/en/
50 mm lens	Canon	https://global.canon/en/
UV-pass filter	–	–

### Experimental model and study participant details

#### Animals

The study species, *Platycnemis phyllopoda*, is a damselfly belonging to the family Platycnemididae and is distributed across Korea, Japan (Tsushima Island),^79^ China (from subtropical south to temperate north), and the Russian Far East (Ussuri region).^80^^,^^81^ In Korea, adults are observed from June to August and primarily inhabit the riparian zones of flowing water bodies. However, they are also widely found in lentic habitats, including reservoirs and ponds that receive inflowing water. Males do not maintain fixed territories. Instead, once ambient light levels increase after sunrise, they become active, moving extensively through vegetation along the water’s edge in search of females. Upon encountering a female, males perform a brief courtship display and then immediately attempt tandem formation-a physical linkage in which the male uses his terminal appendages to clasp the female by her prothorax. Once tandem is established, the male transfers sperm to his secondary genitalia before initiating the copulatory wheel. Following approximately 16 min of copulation,^69^ the male remains in tandem with the female to provide contact guarding during oviposition, a process that typically continues until the afternoon. Tandem ovipositing pairs begin to appear gradually from the morning, reach a peak abundance during the middle of the day (approximately 12:00–14:00), and then decline thereafter. The entire mating process appears to be relatively prolonged, lasting approximately 2–3 h.

### Method details

#### Observation of courtship behavior in *Platycnemis phyllopoda*

Courtship behaviour of male *P. phyllopoda* was recorded on 9 August 2025 between 13:00 and 17:00 at the same study site where the field experiment was conducted (see below). Using a Digital Single-Lens Reflex (DSLR) camera (Nikon D810 equipped with 60 mm and 150 mm lenses), we recorded the courtship behavior of five males. Because naturally occurring courtship interactions were difficult to capture, courtship was elicited by releasing a captured female in front of a perched male using a different female for each male. If the male did not initially detect the female, the female was recaptured and released again in front of the male until a response was elicited. Recording began once the male detected the female and initiated flight. Video recordings captured the sequence from the onset of male flight to the moment the male attempted to form tandem with the female. Courtship behaviour in *P. phyllopoda* was characterised by analysing video recordings played back at one-tenth speed.

#### Scanning electron microscopy

To investigate the microstructure of the expanded tibiae in male *P. phyllopoda*, we utilised five male specimens that had been air-dried at room temperature for over five months. From these individuals, a total of 7 undamaged front-tibiae and 12 undamaged mid- and hind tibiae were excised. The samples were platinum-coated by sputtering without any prior cleaning procedures, using a HITACHI E−1045 sputter coater. As each sample required mounting to expose a single face, separate tibiae were prepared to examine the outer and inner surfaces, respectively. High-resolution imaging was subsequently performed using a field emission scanning electron microscope (FE-SEM; Nova Nano SEM 200, FEI).

#### Measurement of ultraviolet reflectance of expanded tibiae

To determine whether the expanded tibiae of male *P. phyllopoda* exhibit UV reflectance, mature males were photographed at ponds in Mapo-gu, Seoul. Images were captured using a UV-sensitive modified Canon EOS 5D Mark III fitted with a 50 mm lens and a UV-pass filter.

#### Field experiment: Effects of tibial size on male mating success, resighting frequency and observation span

In this experiment, we examined whether the size of the hind tibial expansion in male *P. phyllopoda* predicts mating success, and whether this relationship is affected by weather conditions. On 4 July 2025, we collected all *P. phyllopoda* males observed between 10:30 and 14:00 along a stream in Paju-si, Gyeonggi-do, South Korea. This site was optimal for our experiment because the population was spatially restricted to a stream section of approximately 200 m with accessible riparian zones, thereby maximising the probability of resighting individuals. We collected 84 males and individually marked their wings using paint markers (Uni Posca; Mitsubishi Pencil Co. Ltd, Tokyo, Japan), applying unique combinations of three color dots drawn from five colors (red, yellow, green, blue, and black). Following marking, individuals were held in insect nets (35 × 35 × 60 cm) in the shade areas near the collection site until the paint had fully dried. Preliminary analyses confirmed that wing marking color had no detectable effect on male mating success (Text S1). To measure abdominal length and hind tibial area, each marked male was gently positioned against a Petri dish (diameter: 5.5 cm) and photographed alongside graph paper for scale (Figure 1A) using a smartphone camera (Samsung Galaxy S22). To minimise image distortion, all photographs were taken with the camera positioned perpendicular to the Petri dish. After all males had been photographed, they were released simultaneously at the collection site.

Following release, marked males were monitored daily from 5 July to 13 July 2025. In total, 60 out of 84 marked males were resighted at least once during the monitoring period. Observations were conducted between 10:00 and 15:00, during which the 200 m study reach was traversed back and forth at least five times per day. Upon detection, individuals were photographed using a DSLR camera (Nikon D810 with a 60 mm lens) to allow accurate identification without the need for recapture (Figures 1B–1D). We recorded the daily mating status (single or paired with a female) of each marked male found. Given that paired individuals were generally active between 09:00 and 18:00, and mate guarding typically lasted 2–3 h (I. Yang, personal observation), this observation window was deemed sufficient to detect most mating pairs. A male’s daily mating status was classified as 'paired' if he exhibited tandem formation at least once during the daily observation period; otherwise, he was recorded as 'single'.

To validate the use of a single daily mating status rather than recording status per observation, we analyzed instances of repeated daily sightings. Out of 67 instances where a male was observed more than twice in a single day, mating status changed in 19 instances (single to paired: 13 instances; paired to single: 6 instances). Furthermore, no males were observed in copula for more than 3 h (I. Yang, personal observation). Importantly, there were no instances where a male’s mating status changed from paired to single and back to paired within the same day. This indicates that males neither remain in tandem throughout the entire day nor immediately secure a new mate after copulation ends. Consequently, assigning a single daily mating status—where a male was considered 'paired' for the day even if found with a female on only one out of multiple observations—provides a robust representation of his daily reproductive success.

To analyze the effect of weather conditions on the relationship between hind tibial area and male mating success, we obtained meteorological data (specifically air temperature, cloud cover, and UV index) from the Korea Meteorological Administration (data.kma.go.kr). No rainfall occurred during the monitoring period. Because male mating status was assessed on a daily basis (as described above), we calculated daily meteorological variables by averaging the hourly data recorded between 09:00 and 15:00 for each day.

Morphological measurements were based on photographs of individual males and were conducted using ImageJ software (version 1.54g). Abdominal length was measured from the second to the tenth segment. Regarding tibial area, we focused exclusively on the hind tibiae because the mid tibiae of live individuals could not be reliably positioned flush against the Petri dish. Consequently, the area of one randomly selected hind tibia was measured for each individual (Figure 1A). If an individual’s posture prevented a specific tibia from being photographed horizontally, measurements were taken from the alternative hind tibia that was flush against the surface. One Individual for which neither hind tibia met these criteria were excluded from the analysis. Tibial area was quantified in ImageJ by applying a threshold based on the contrast between the white tibia and the black background. Once the tibial region was detected, its total area was automatically calculated, ensuring that any internal dark stains were included in the final measurement.

#### Allometric slope

To calculate the precise allometric patterns of *P. phyllopoda*, we collected 47 males on 28 July 2025 from a population in Yangpyeong-gun, Gyeonggi-do, South Korea, located approximately 80 km from the field experiment site. These individuals were subsequently transferred to the laboratory for morphometric analysis. The following day, individuals were euthanised by freezing for approximately 30 min, after which their appendages were dissected. High-resolution images were captured using a stereomicroscope (Leica S9D) to measure the following traits: (1) abdominal length (segments 2–10), (2) right hindwing length (from nodus to pterostigma), (2) fore tibial length, (3) mid tibial length, (4) hind tibial length, (5) mid femur length, (6) hind femur length, (7) mid tibial area, and (8) hind tibial area. For all tibial measurements, the mean of the left and right legs was calculated; however, if one leg was damaged, the length of the single available tibia was used. Dissected appendages were photographed as flat as possible against the surface to minimise distortion. All measurements were performed in the program ImageJ (version 1.54g) following the same protocols described in the above. Preliminary analyses confirmed that hind tibial allometry estimated was consistent across populations under ordinary least squares (OLS) regression, whereas significant differences in scaling were observed under the standardised major axis (SMA) regression when comparing preserved specimens (Yangpyeong population) and live individuals used in the field experiment (Paju population) (Yangpyeong population: OLS, 0.72 (0.42, 1.02); SMA, 1.27 (0.99, 1.62), Paju population: OLS, 0.76 (95%CI: 0.33, 1.19); SMA, 2.12 (95%CI: 1.72, 2.61), Text S2).

### Quantification and statistical analyses

#### Field experiment

Because all weather variables (air temperature, cloud cover, and UV index) were strongly correlated during the observation period, we conducted a principal component analysis (PCA) using the prcomp function in the *stats* package (v.4.5.0) in R (v. 4.5.0)^82^ to reduce them into a single variable. The first principal component (PC1) explained 69% of the total variance and was characterised by higher temperature and UV levels but lower cloud coverage (Table S1). We refer to this as “weather PC” (Table S1) and included it as a covariate in the model.

To test the relationship between tibia size and daily mating success, we used a generalised linear mixed-effects model (GLMM) with binomial errors, fitting mating success as the response variable (0 = pairing failed, 1 = pairing succeeded). The model included the following fixed effects: loss of the expanded tibia (0 = intact, 1 = lost), z-standardised observation day, z-standardised and log-transformed hind tibial area, z-standardised and log-transformed abdomen length (as a body size index) and the weather PC. Furthermore, we deliberately restricted our interaction terms solely to the interaction between weather PC and hind tibia area. This decision was strictly hypothesis-driven: testing this specific interaction was a primary objective of our study, whereas including all possible two-way interactions lacked theoretical justification and would have unnecessarily overparameterised the models. In addition, because the daily mating status of each male was measured repeatedly over the observation period, male identity was fitted as a random effect. Finally, formal model diagnostics confirmed that overdispersion was not an issue for this binary response model.

In addition, we tested whether tibia size was associated with resighting frequency (the total number of resighting days; min = 0, max = 9) or observation span (the number of days between the first and last sightings, inclusive; min = 1, max = 10). Having confirmed overdispersion in the data, we fitted negative binomial models (family = nbinom2). In these models with resighting frequency or observation span as the response variable, z-standardised and log-transformed abdomen length (body size index) and hind tibial area were included as covariates.

All generalised linear (mixed-effects) models were fitted using the glmmTMB package (v. 1.1.13)^83^ in R (v. 4.5.0). We found no evidence of multicollinearity among predictors (all variance inflation factors <1.6^84^) as assessed using the *performance* package.^85^ All models were checked for overdispersion and zero-inflation using the *DHARMa* package (v. 0.4.7).^86^

#### Allometry

We estimated the allometric relationship using the equation log(Y) = log(α) + βlog(X), where Y represents the size of the focal trait (e.g., trait length or square root transformed area), X is the body size index (abdomen length), α is the allometric intercept, and β represents the allometric slope. Positive and negative allometry was determined by whether the estimated allometric slope was significantly greater or less than 1, respectively. To estimate allometry, we employed the two most widely used regression approaches: ordinary least squares (OLS) and standardised major axis (SMA) regressions.^56^^,^^87^^,^^88^^,^^89^ These methods differ fundamentally in their assumptions regarding measurement error. OLS regression assumes that the predictor variable (body size) is measured without error, whereas SMA accounts for measurement error in both the predictor and dependent variables.^89^^,^^90^ Because biological measurements inevitably contain some error, OLS tends to mathematically underestimate allometric slopes. In our study, we actively sought to minimise measurement error by analysing high-resolution macro images and utilising multiple body size indices (abdomen length and hindwing length) during initial assessments. Our measurement methodology demonstrated exceptionally high repeatability (see Text S3). While such high precision theoretically reduces the downward bias inherent in OLS regressions,^88^ the ongoing debate regarding the optimal regression approach for allometry necessitates a rigorous analytical approach.^88^^,^^91^^,^^92^^,^^93^ Thus, given the inherent measurement errors associated with morphological traits, we prioritised the interpretation of allometric slopes derived from the SMA approach—which accounts for error variance in both variables—while providing OLS estimates as supplementary information for comparative purposes (Text S4). OLS regressions were fitted using the *glmmTMB* package,^83^ and SMA regressions were performed using the *smatr* package (v. 3.4.8)^94^ in R (v. 4.5.0).

In our allometric analyses, we used abdomen length (from segment 2 to 10) as an index of overall body size because it can be measured accurately and constitutes more than half of the total body length. Although hindwing length is frequently used as a body size proxy in studies of damselflies and dragonflies,^95^^,^^96^^,^^97^^,^^98^ our field methodology precluded its use. Specifically, because we photographed the ventral side of the males in the field to precisely estimate hind tibial area, the nodus was obscured, making accurate hindwing measurements impossible. Consequently, we adopted abdomen length as our primary size index for the field data.

To validate this alternative metric, we utilised images of specimens used to calculate the precise allometric patterns in the laboratory (see above) to compare allometric slopes calculated using both abdomen length and hindwing length. The slopes derived from the abdomen length index were highly consistent with those derived from the hindwing length index (see Table 3). Therefore, to maintain methodological consistency, we uniformly applied abdomen length as the standard index of overall body size across all allometric analyses for both field and laboratory datasets.
