# Supplementary material for: Expanded tibiae enhance male mating success in the damselfly Platycnemis phyllopoda
Source: iScience. 2026 Jul 10;29(8):116759. doi: 10.1016/j.isci.2026.116759 (PMC13380454; doi:10.1016/j.isci.2026.116759)
Supplement: Document S1. Text S1–S4 and Tables S1 and S2 [file mmc1.pdf]

iScience, Volume *29*

Supplemental information

Expanded tibiae enhance male mating success in the damselfly *Platycnemis phyllopoda*

Ilju Yang and Chang S. Han

Supplementary information

Expanded tibiae enhance male mating success in the damselfly *Platycnemis phyllopoda*

Ilju Yang [1] and Chang S. Han [1,2,3*]

[1] Department of Biology, Kyung Hee University, Seoul 02447, Korea

[2] Korea Institute of Ornithology, Kyung Hee University, Seoul 02447, Korea

[3] Lead contact

* Correspondence: hcspol@gmail.com

Text S1. Effects of colour marking on male mating success

To evaluate whether the colour markings utilised for individual identification affected male mating success, we employed a generalised linear mixed-effects model (GLMM) with a binomial error distribution and logit link function. The GLMM was fitted using the *glmmTMB* package [64] in R (version 4.5.0). Mating success was included as the binary response variable (0 = failed pairing, 1 = successful pairing). The presence or absence of each specific marking colour (red, blue, black, green, and yellow) was included as a fixed effect (0 = absent, 1 = present). In the model, male identity was fitted as a random effect to account for repeated observations. This analysis revealed that the presence of colour dots on the wings did not significantly affect male mating success for any of the colours tested (red: β(SE)=0.12(0.36), z=0.33, P=0.74; yellow: β(SE)=-0.10 (0.39), z=-0.25, P=0.81; green: β(SE)=-0.55(0.38), z=-1.44, P=0.15; blue: β(SE)=0.67(0.42), z=1.61, P=0.11; black: β(SE)=0.12(0.44), z=0.27, P=0.79).

Text S2. Differences in allometric slopes for hind tibial area between Paju and Yangpyeong populations.

While the ordinary least squares (OLS) slopes were comparable between the two regions (0.76 vs 0.72), Standardised major axis (SMA) analysis revealed that the Paju population (2.12) exhibited a much stronger positive allometry than the Yangpyeong population (1.27). This difference might stem from differences in trait variance between the two sites. In Paju, where field experiments were conducted, the variance for log-transformed abdomen length was 2.73×10^{-4}, while that for log-transformed tibial area was 2.52×10^{-3}. In contrast, in Yangpyeong, where more controlled allometric assessments were performed, the variances were 3.40×10^{-4} for abdomen length and 1.28×10^{-3} for tibial area. Consequently, as the SMA slope is fundamentally determined by the ratio of the variances of the two traits, the substantially higher variation in tibial area relative to body size in Paju resulted in a steeper SMA estimate.

These differences in variance may have been influenced by the respective measurement techniques. In Paju, individuals were measured in the field using a smartphone camera by pressing the specimen against a Petri dish, ensuring the tibiae were reliably positioned flush against the surface. In contrast, for the Yangpyeong population, the legs were detached and measured under laboratory conditions. It is highly probable that the former method introduced greater measurement noise. Given that the error associated with abdomen length was considerably lower than that for area measurements (see Supplementary Text S3), we cannot rule out the possibility of the impact of noise in the area data on the SMA slope**.**

Specifically, the increased noise in the Paju field data likely depressed the correlation coefficient (r) between the two traits, whereas the reduced noise in the Yangpyeong dataset likely yielded a higher r. Consequently, even when OLS allometric slopes were similar between two populations, the SMA analysis revealing a much more positive scaling relationship in the Paju population.

Text S3. Repeatability of morphological measurements

To evaluate the precision of our measurement methods, we performed repeated estimations of male abdomen length and the areas of the mid- and hind-tibiae (including both the left and right tibiae) from 20 males.

We quantified the repeatability (i.e., intra-observer error) of our measurements using two distinct approaches to capture different sources of potential variance. First, we estimated intra-observer error occurring during the digital measurement process ("digital measurement error"); for this, we measured the abdomen length or tibial area from a single image three times using ImageJ based on standardised criteria. Second, we assessed the error associated with sample orientation, physical handling and image acquisition ("procedural and positioning error"). This involved independently repositioning the same sample on the microscope stage three times, with a new photograph taken after each placement. These subsequent images were then used to quantify the respective lengths or areas.

To calculate measurement repeatability, we employed linear mixed-effects models with z-transformed and log-transformed data as response variables. For abdomen length, male identity was included as a random effect. For tibial area, the model included a combination of male identity and tibial side (left vs right) as a random effect. The models were fitted using the *glmmTMB* package [64] in R (version 4.5.0).

For the first type of the measurement error, the repeatabilities were 0.94 and 0.96 for the mid and hind tibial areas, respectively, and 0.96 for abdomen length. These high values indicate that the structures were unambiguously distinguished from the background during digital tracing. For the second type of the measurement error, the repeatabilities were 0.84 and 0.88 for mid and hind tibiae areas, respectively, and 0.95 for abdomen length. This demonstrates that the variance introduced by positioning the specimen on the microscopy stage was minimal. In addition, the measurement error associated with physical handling was considerably lower for abdomen length compared to tibial area, likely due to the more straightforward linear measurement of the abdomen. Consequently, all repeatability values exceeded the generally accepted thresholds for biological measurements, confirming the high reliability of our dataset.

Text S4. Allometric analyses using the ordinary least squares (OLS) approach

Consistent with theoretical expectations and existing literature, the allometric slopes estimated via the OLS approach were lower than those derived from the SMA framework. Under the OLS approach, the lengths of non-sexual traits—the fore tibia, mid tibia, hind tibia, mid femur, and hind femur —showed negative allometry (Table S2). The mid tibial area showed slightly positive allometry, whereas the hind tibial area exhibited slightly negative allometry (Table S2). However, neither of these OLS slopes significantly differed from isometry (Table S2).

Table S1. Principal component (PC) analyses of weather characteristics.

Weather characteristics	PC1
Temperature	0.67
Cloud	-0.33
UV	0.66
Descriptive statistics	
Standard deviation	1.43
Proportion of variance	0.69

Table S2. Allometric slopes for non-sexual traits (lengths of the fore tibia, mid tibia, hind tibia, mid femur, and hind femur) and sexual traits (mid and hind tibial areas) in male *Platycnemis phyllopoda*. Slopes were estimated using ordinary least squares (OLS) regressions, with abdomen and hindwing length serving as body size proxies. Area values were square-root transformed prior to analysis to ensure dimensional consistency. Values represent slope estimates with 95% confidence intervals, and sample sizes (N) are provided for each trait.

Body size proxy: Abdomen	OLS slope (95% CI)	Body size proxy: Hindwing	OLS slope (95% CI)
Hindwing (N=47)	0.76 (0.56, 0.96)	Abdomen	0.72 (0.53, 0.90)
Fore tibia (N=46)	0.55 (0.31, 0.80)	Fore tibia	0.63 (0.40, 0.86)
Mid tibia (N=47)	0.52 (0.28, 0.77)	Mid tibia	0.57 (0.34, 0.79)
Hind tibia (N=47)	0.47 (0.20, 0.74)	Hind tibia	0.51 (0.26, 0.77)
Mid femur (N=47)	0.62 (0.37, 0.88)	Mid femur	0.65 (0.41, 0.89)
Hind femur (N=37)	0.43 (0.22, 0.65)	Hind femur	0.43 (0.23, 0.64)
Mid tibial area (N=47)	1.20 (0.86, 1.53)	Mid tibial area	1.09 (0.75, 1.44)
Hind tibial area (N=47)	0.72 (0.42, 1.02)	Hind tibial area	0.72 (0.43, 1.00)

Supplementary Video S1. Courtship behaviour of *Platycnemis phyllopoda*. In the final stage, the female rejects the male by arching her abdomen upwards.

Supplementary Video S2. Courtship behaviour of *Platycnemis phyllopoda*. The female remains stationary and accepts the attempt at tandem formation.
